# Proteases secreted by *Trichinella spiralis* intestinal infective larvae damage the junctions of the intestinal epithelial cell monolayer and mediate larval invasion

**DOI:** 10.1186/s13567-022-01032-1

**Published:** 2022-03-07

**Authors:** Yan Yan Song, Qi Qi Lu, Lu Lu Han, Shu Wei Yan, Xin Zhuo Zhang, Ruo Dan Liu, Shao Rong Long, Jing Cui, Zhong Quan Wang

**Affiliations:** Department of Parasitology, Medical College, Zhengzhou University, Zhengzhou, 450052 China

**Keywords:** *Trichinella spiralis*, intestinal infective larvae, serine proteases, cysteine proteases, gut epithelium, invasion

## Abstract

The intestinal epithelium is the first natural barrier against *Trichinella spiralis* larval invasion, but the mechanism of larval invasion of the gut epithelium is not fully elucidated. The aim of this study was to investigate whether the excretory/secretory proteins (ESPs) of *T. spiralis* intestinal infective larvae (IIL) degrade tight junction (TJ) proteins, to assess the main ESP proteases hydrolysing TJ proteins using various enzyme inhibitors and to define the key invasive factors in IIL invasion of the gut epithelium. The results of immunofluorescence, Western blot and Transwell assays showed that serine proteases and cysteine proteases in the ESPs played main roles in hydrolysing occludin, claudin-1 and E-cad and upregulating claudin-2 expression. Challenge infection results showed that IIL expulsion from the gut at 12 hpi was significantly higher in mice which were infected with muscle larvae (ML) treated with a single inhibitor (PMSF, E-64, 1,10-Phe or pepstatin) or various mixtures containing PMSF and E-64 than in mice in the PBS group or the groups treated with an inhibitor mixture not containing PMSF and E-64 (*P* < 0.0001). At 6 days post-infection, mice which were infected with ML treated with PMSF, E-64, 1,10-Phe or pepstatin exhibited 56.30, 64.91, 26.42 and 31.85% reductions in intestinal adult worms compared to mice in the PBS group (*P* < 0.0001). The results indicate that serine proteases and cysteine proteases play key roles in *T. spiralis* IIL invasion, growth and survival in the host and that they may be main candidate target molecules for vaccines against larval invasion and development.

## Introduction

*Trichinella spiralis* is an important zoonotic parasitic nematode of the genus *Trichinella*. *T. spiralis* infection is caused by the ingestion of raw or poorly cooked animal meat infected with encapsulated infective muscle larvae (ML). Adult worms (AWs) and ML lodge in the small intestine and skeletal muscles of a host. Following ingestion, the encapsulated ML in meat are liberated from their collagen capsules with the help of digestive fluids and activated into intestinal infective larvae (IIL) after coming into contact with gut contents or bile [1, 2]. IIL invade intestinal epithelial cells (IECs) and develop into the AW stage after undergoing four molts at 31 h post-infection (hpi). Pregnant AWs deposit newborn larvae (NBL), which migrate via the lymphatic and blood systems to the skeletal muscles, where they develop into encapsulated ML to complete the lifecycle [3, 4]. The gut epithelium is the first natural barrier against IIL intrusion and the principal site of interactions between the host and the nematode *T. spiralis* [5]. Nevertheless, the mechanism of IIL intrusion into the intestinal epithelium has not been fully elucidated [6, 7]. As *T. spiralis* larvae do not have oral appendices or a spike, intrusion of the gut epithelium is unlikely to be due to larval mechanical penetration; instead, it might be due to the degradation and destruction of the gut epithelium by various proteases in the excretory/secretory proteins (ESPs) of IIL [8–10]. The proteases in IIL ESPs are directly exposed to and contact the gut mucosa, and they might mediate IIL penetration of the gut epithelium [11, 12].

Previous studies have shown that when IIL are co-cultured with IEC monolayers, the IIL invade the monolayer and generate various kinds of proteases that pass into the IECs [13, 14]. The proteases secreted by intestinal parasites disrupt gut epithelial integrity and participate in parasite invasion of the gut mucosa [15–17]. The mucus layer secreted by intestinal goblet cells and gut epithelial columnar cells forms the native mechanical defence at the mucosal surface. Gut mucosal permeability is regulated by tight junctions (TJs) localized below the microvilli of adjacent epithelial cells. TJs are composed of claudins, adherens junctions, desmosomes and keratin filaments. Mucin and TJ proteins can be degraded and destroyed by diverse proteases produced by intestinal parasites. Serine proteases produced by the murine gut nematode *Trichuris muris* degrade the intestinal mucus barrier [18]. A cathepsin B-like (gCatB) enzyme produced by *Giardia duodenalis* trophozoites induces the degradation of the TJ proteins occludin and claudin-1 in the gut epithelial cell monolayer [19]. A recombinant cysteine protease from *Spirometra erinaceieuropaei* plerocercoid (rSeCP) hydrolyses fibronectin and collagen I/IV in vitro [20]. An *Angiostrongylus cantonensis* cathepsin B-like protease (Ac-cathB-1) has the capability to degrade fibronectin, laminin and the extracellular matrix of IEC-6 monolayers, and inhibition of Ac-cathB-1 enzymatic activity with antiserum partly impedes larval penetration of the gut [21]. Our previous studies demonstrated that anti-ESP immune serum inhibited *T. spiralis* invasion of IECs and larval development, suggesting that the ESPs might contain IEC invasion-related proteases [22]. *T. spiralis* aspartic protease in the ESPs cleaves several host proteins and facilitates larval invasion of IECs [23]. Specific binding of *T. spiralis* elastase-1 (TsEla) with host IECs promotes larval intrusion into IECs and the gut mucosa [24].

The aim of the present study was to investigate the degradation of and damage to the tight junctions (TJs) of IEC monolayers by *T. spiralis* IIL ESPs, to assess the main kinds of natural proteases with enzymatic activity in the ESPs using various enzyme inhibitors and to define the key IIL invasive factors for invasion of the gut epithelium. The various specific protease inhibitors used in this study included the serine protease inhibitor phenylmethylsulfonyl fluoride (PMSF), cysteine protease inhibitor E-64, aspartic protease inhibitor 1,10-phenanthrolin (1,10-Phe), metalloprotease inhibitor pepstatin A (pepstatin) and ethylenediaminetetraacetic acid (EDTA).

## Materials and methods

### Parasites and experimental animals

A *Trichinella spiralis* (ISS534) strain was isolated from a naturally infected pig in Henan Province, China. This strain was passaged in BALB/c mice in our department every 6 months. Female BALB/c mice (6–8 weeks old) were obtained from the Experimental Animal Center of Zhengzhou University (No. SCXK 2017-0001).

### Collection of IIL and their ESPs

ML were obtained by artificially digesting the carcasses of mice infected with *T. spiralis* at 42 days post-infection (dpi) [25]. IIL were collected from *T. spiralis*-infected murine intestines at 6 hpi [26]. IIL ESPs was prepared as described previously [27, 28]. Briefly, after thorough washing with sterile physiological saline and serum-free RPMI 1640 medium (100 U penicillin/mL and 0.1 mg/mL streptomycin), IIL worms were cultured under the conditions of 5000 worms/mL of medium at 37 ℃ and 5% CO_2_ for 18 h. The supernatant containing IIL ESPs was concentrated at 4 ℃ and 5000 × *g* with an Amicon Ultra-3 Centrifugal Filter Unit (MW cut-off: 3 kDa).

### Gut epithelial cell culture

The human colonic epithelial cell line Caco-2 was purchased from the Cell Resource Center of the Shanghai Institute for Biological Sciences of the Chinese Academy of Sciences. Caco-2 cells were cultured and maintained in Modified Eagle’s Medium (MEM)-ALPHA (Sigma–Aldrich, USA) supplemented with 10% foetal bovine serum (FBS) (Gibco), 100 U/mL penicillin, 0.1 mg/mL streptomycin and 100 mM non-essential amino acids (Solarbio, Beijing, China). Cells were seeded in T25 flasks (NEST, Wuxi, China) and cultured for 7 days. After being digested and isolated, the cells were cultured for 14–21 days to confluence on glass coverslips in 6-well plates. For all cell cultures, the medium was replaced two times weekly, and the cells were incubated at 37 ℃ in a 5% CO_2_ incubator [29, 30].

### In vitro larval invasion of Caco-2 cells

To investigate the inhibitory effects of various protease inhibitors on IIL invasion of the intestinal epithelium, in vitro IIL intrusion into a Caco-2 cell monolayer was performed as previously reported [31, 32]. Briefly, ML were first activated with 5% mouse bile for 2 h at 37 °C to generate IIL [33]. The IIL were pre-incubated at 37 °C for 2 h with various doses of different inhibitors (1.25 mM PMSF, 24 μM E-64, 2.4 mM 1,10-Phe or 10 μM pepstatin) alone or in combination. The inhibitor stock solutions were made with the anhydrous solvent dimethyl sulfoxide (DMSO), and these solutions were diluted before use in the final cell medium (MEM). The final dilution of DMSO in the cell medium that was used as a negative or basal control was 0.1%. Then, 100 IIL treated with inhibitors were added to a cell monolayer and cultured in semisolid MEM-ALPHA (serum-free MEM and 1.75% agarose) at 37 °C and 5% CO_2_ for 2 h [34]. Larval invasion of the cell monolayer was examined and quantified under an inverted phase-contrast microscope [35]. The invaded worms were active and migratory in the monolayer and had a remarkable migratory path, whereas non-invaded worms were crimped on the surface of the monolayer [12]. The inhibition rate for larval invasion was calculated based on the mean number of invaded larvae in the inhibitor group compared with that in the PBS control group using the following formula: inhibition rate (%) for larval invasion = (mean number of invaded larvae in the PBS control group—mean number of invaded larvae in the inhibitor group)/mean number of invaded larvae in the PBS control group × 100%. To observe the larval migratory path, the agarose was removed, and the coverslips were washed twice with MEM-10% FBS. The cells were stained with the fluorescent nuclear dye propidium iodide (PI; 0.03 mg/mL in MEM-10% FBS) at 4 °C for 30 min. After being washed twice with MEM-10% FBS, the cells were fixed in 4% paraformaldehyde for 30 min, washed in PBS, permeabilized and blocked in a blocking solution (PBS containing 0.1% Triton X-100 and 5% normal goat serum) at room temperature (RT) for 20 min. The monolayers were probed with mouse immune serum against IIL ESPs (1:100) at 37 °C for 1 h and then incubated with FITC-conjugated goat anti-mouse IgG (1:100, Servicebio, Wuhan, China) at RT in the dark for 40 min. Finally, the coverslips were mounted on glass slides and examined under a fluorescence microscope [36].

### Indirect immunofluorescence test (IIFT)

To identify which kinds of proteases in IIL ESPs degrade the TJ proteins of the gut epithelium, 20 μg/mL ESPs was pre-treated at 37 °C for 2 h with various inhibitors (1.25 mM PMSF, 24 μM E-64, 2.4 mM 1,10-Phe or 10 μM pepstatin) or various inhibitor mixtures (PMSF + E-64 + 1,10-Phe + pepstatin, E-64 + 1,10-Phe + pepstatin, PMSF + 1,10-Phe + pepstatin, PMSF + E-64 + pepstatin or PMSF + E-64 + 1,10-Phe). Caco-2 cells were grown to confluence on glass coverslips in MEM [37], and the cell monolayers were incubated with treated ESPs at 37 °C for 2 h. Normal Caco-2 cells treated with 0.1% DMSO were used as a negative control, and 20 μg/mL ESPs without inhibitors was used to assess the hydrolysis of TJ proteins. After being washed with PBS, the monolayers were fixed with 4% formaldehyde for 20 min. After washing, the monolayers were permeabilized for 10 min with 0.25% Triton X-100 saponin in PBS and blocked with 1% bovine serum albumin (BSA; Sigma, USA). The monolayers were probed overnight at 4 °C with rabbit anti-human polyclonal antibodies against occludin (1:160), claudin-1 (1:16), and claudin-2 (1:125) (Thermo Fisher, USA) or a rat anti-human monoclonal antibody against E-cad (1:100; Santa Cruz, USA) and then incubated with Cy3-conjugated goat anti-rabbit IgG (1:100) or FITC-conjugated goat anti-rat IgG (1:100; Servicebio) for 1 h at 37 °C. Finally, the monolayers were incubated for 5 min with 4′,6-diamidino-2-phenylindole (DAPI) to specifically stain cell nuclei [38, 39]. Upon completion of the staining procedure, the coverslips were transferred to glass slides and observed with fluorescence microscopy. Moreover, the cellular localization of TJ proteins was further examined under a fluorescence microscope and analysed with Olympus Fluoview software [40, 41].

### Western blot analysis

Caco-2 cells were cultured in MEM-10% FBS. After being washed fully with PBS, the cell monolayers were incubated at 37 °C for 2 h in serum-free MEM with 20 μg/mL IIL ESPs pre-treated with various inhibitors (1.25 mM PMSF, 24 μM E-64, 2.4 mM 1,10-Phe or 10 μM pepstatin) alone or in mixtures. Normal Caco-2 cells treated with 0.1% DMSO were used as a negative control, and 20 μg/mL ESPs without inhibitors was used to assess the hydrolysis of TJ proteins. The cells were treated with a rubber cell scraper and mixed fully [42]. The cells were lysed in RIPA buffer (40 mM Tris–HCl pH 7.6, 150 mM NaCl, 2 mM EDTA, 10% glycerol, 1% Triton X-100, 0.2% SDS, 1 mM PMSF and a protease inhibitor cocktail), ultrasonicated in an ice bath for 30 s and centrifuged at 12 000 *g* for 15 min to remove any cell fragments. The cell protein concentration was measured with a Bradford assay. The soluble proteins from the cells were separated by 10% SDS–PAGE [43, 44]. The proteins were transferred to PVDF membranes (Millipore, USA). The membranes were blocked with 5% non-fat milk diluted in TBST at 37 °C for 2 h and cut into strips. The strips were probed using antibodies against occludin (1:500), claudin-1 (1:200), claudin-2 (1:200) or E-cad (1:100) (ThermoFisher, USA) overnight at 4 °C, and an anti-GAPDH antibody (1:1000) was used as an internal control [45]. The strips were incubated with HRP-conjugated anti-rabbit IgG or HRP-conjugated anti-rat IgG (1:5000; Southern Biotech, USA) at 37 °C for 1 h. Three washes with TBST were performed after each incubation. The strips were developed with an enhanced chemiluminescence kit (CWBIO, Beijing, China) [46, 47]. The optical density of each strip was measured with a scanner and quantified with ImageJ software [48]. The corresponding GAPDH expression levels were used as internal controls, and the results are expressed as a percentage relative to the normal Caco-2 cell control group.

### Transwell assay

**Figure 1:**
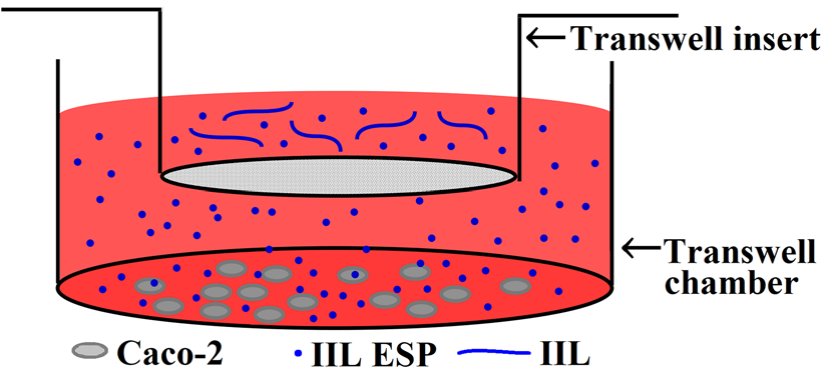
**Transwell experimental system used for identifying direct interactions between the main IIL ESP proteases and Caco-2 cells.** Caco-2 cells were cultured on glass coverslips in a 12-well Transwell chamber, and 200 IIL pre-treated with various inhibitors were added to the insert in serum-free MEM medium and incubated at 37 ℃ for 2 h. The inhibitor doses used in the Transwell assay were 1.25 mM PMSF, 24 μM E-64, 2.4 mM 1,10-Phe and 10 μM pepstatin. There was a filter membrane on the inserted sieve to prevent IIL from invading and destroying the Caco-2 cell monolayer, but ESPs could pass through the insert and directly contact the monolayer, thus affecting epithelial TJ proteins.

To investigate epithelial barrier breakdown by the main proteases in the ESPs secreted by IIL, a Transwell assay was performed according to the method in a publication on the interaction between the *Giardia intestinalis* secretome and IECs [49]. Briefly, glass coverslips were first placed in a 12-well Transwell system, and Caco-2 cells were grown to confluence on the glass coverslips in MEM-10% FBS. IIL were first pre-treated at 37 °C for 2 h with various inhibitors (1.25 mM PMSF, 24 μM E-64, 2.4 mM 1,10-Phe or 10 μM pepstatin) alone or in mixtures, as described in the IIFT subsection. Normal Caco-2 cells treated with 0.1% DMSO were used as a negative control, and normal IIL not treated with inhibitors were used to assess the ESP-mediated hydrolysis of TJ proteins. After being washed completely using PBS, two hundred IIL treated with inhibitors were added to the Transwell insert, which was placed in wells containing serum-free MEM and incubated at 37 °C for 2 h. Since the proteases in IIL ESPs could hydrolyse FBS in MEM and to avoid FBS hampering the hydrolysis of cell monolayer-expressed TJ proteins by IIL-secreted proteases, serum-free MEM was used when the IIL were co-cultured with Caco-2 cells in the Transwell system. As shown in Figure 1, the Caco-2 cell monolayer was directly exposed to IIL ESPs through the Transwell insert. After being washed using PBS, the glass coverslips with adhered Caco-2 cells were fixed, blocked, probed, stained and examined under a fluorescence microscope as described above.

### SDS–PAGE analysis

To determine the key proteases in IIL ESPs involved in degrading host proteins, collagen I and fibronectin were used as the hydrolysis-sensitive substrates of proteases in IIL ESPs. Various doses of IIL ESPs (5.50, 8.25, 11.00, 13.75, and 16.50 μg) were first pre-treated at 37 °C for 30 min with various single inhibitors (1 mM PMSF, 10 mM EDTA, 20 μM E-64 or 10 μM pepstatin) or different inhibitor mixtures (EDTA + PMSF + E-64, EDTA + PMSF + pepstatin, EDTA + E-64 + pepstatin, PMSF + E-64 + pepstatin, or EDTA + PMSF + E-64 + pepstatin). A protease inhibitor cocktail (PMSF + E-64 + pepstatin + leupeptin + aprotinin + bestatin; BBI, Canada) was also used in this assay. Then, the ESPs treated with the various inhibitors were incubated with collagen I (4.5 µg) and fibronectin (5 µg) (Sigma, USA) at different pH values (4.5, 5.5, 6.5, and 7.5) overnight at 37 °C. The reactions were stopped by the addition of sample buffer containing 2% SDS and 1% β-mercaptoethanol [20, 23, 30]. IIL ESPs not treated with inhibitors were also used to hydrolyse collagen I and fibronectin, whereas IIL ESPs inactivated at 100 °C for 10 min were used as a negative control for proteases. The total volume of the mixture was 27.5 μL. All samples were denatured at 100 °C for 5 min, and 20 μL of each sample was analysed by SDS–PAGE analysis with 5% stacking gels and 12% resolving gels and Coomassie brilliant blue R-250 staining [50].

### Challenge infection with inhibitor-treated *T. spiralis* larvae

To evaluate larval infectivity (invasive capacity), survival and development in the host gut following inhibitor treatment, *T. spiralis* ML were pre-incubated at 37 °C for 2 h with various inhibitors (1.25 mM PMSF, 24 μM E-64, 2.4 mM 1,10-Phe or 10 μM pepstatin) alone or in mixtures (PMSF + E-64 + 1,10-Phe + pepstatin, E-64 + 1,10-Phe + pepstatin, PMSF + 1,10-Phe + pepstatin, PMSF + E-64 + pepstatin or PMSF + E-64 + 1,10-Phe). ML treated with 0.1% DMSO or PBS were used as a negative or normal control. A total of 110 mice were divided into 11 groups (10 mice per group). Each mouse was infected orally with 300 ML treated with an inhibitor or mixture [4, 41]. The expelled IIL in faeces were collected within 12 h following challenge and counted as described previously [51]. The following formula was used: larval expulsion rate (%) = (mean number of expelled IIL in faeces/mean number of inoculated ML) × 100%. The infected mice were sacrificed at 6 dpi, intestinal adult worms were recovered and counted, and the reductions in adult worms between the inhibitor-treated group and the PBS control group were ascertained [52].

### Statistical analysis

SPSS 22.0 software was used for all the data analyses in this study, and the data for larval invasion, protein expression levels, expelled IIL and intestinal adult burden are presented as the mean ± standard deviation (SD). After being tested by Shapiro–Wilk’s test and Levene’s test to check the normality and homogeneity of the data, one-way ANOVA and Student’s *t* test were used to analyse inter-group differences. The correlation between inhibitor dose and larval invasion was compared with Spearman’s rank correlation (*r*) analysis. *P* < 0.05 was regarded as statistically significant.

## Results

### Inhibitory effects of various inhibitors on IIL invasion of Caco-2 cell monolayers

**Figure 2:**
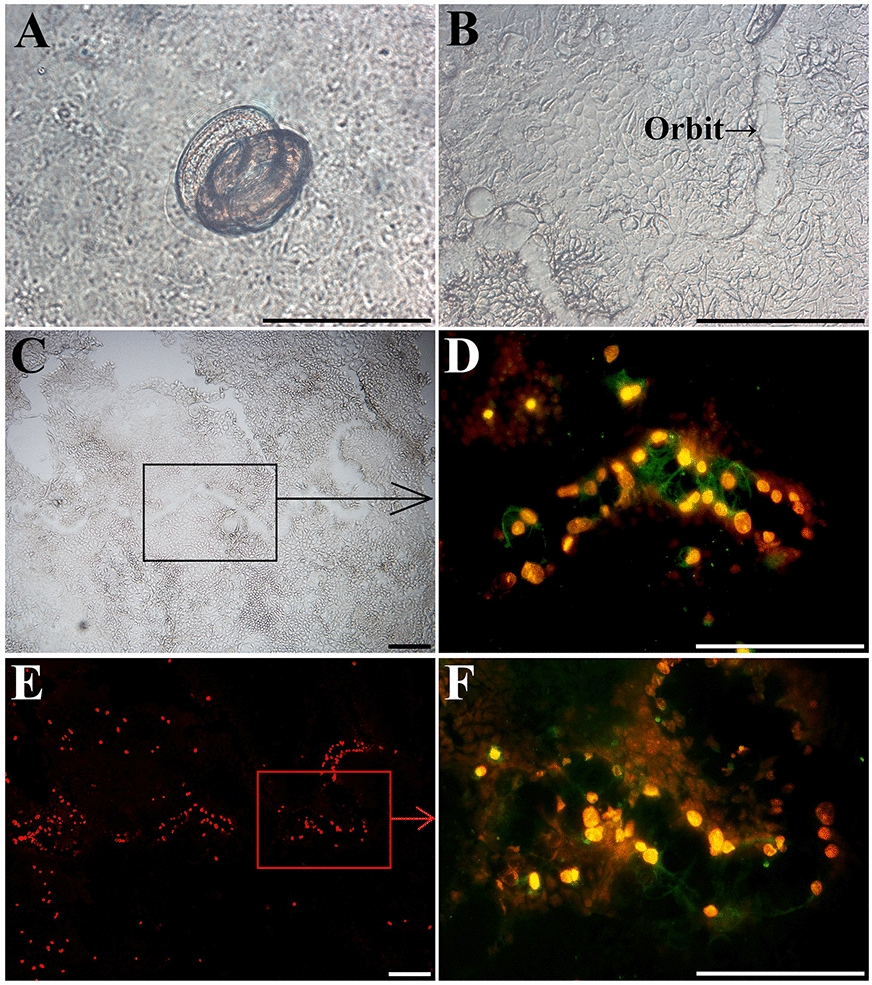
**T. spiralis IIL invasion and migration in a Caco-2 cell monolayer.** After a Caco-2 cell monolayer was covered with semisolid MEM containing IIL and cultured at 37 °C for 2 h, IIL invasion and migration were examined by microscopy. **A** Non-invaded larvae were coiled on the surface of the Caco-2 cell monolayer. **B**, **C** Invaded larvae left a migratory path in the monolayer. **D** The nuclei of dead cells were dyed orange with PI, IIL ESPs were recognized with anti-ESP immune serum, and green fluorescence in the cytoplasm of damaged cells was observed by fluorescence microscopy. **E** The nuclei of damaged cells were stained intensely and uniformly orange by PI, showing the serpentine trail left by the parasite. **F** The IIL ESPs within the migratory path were detected using anti-ESP immune serum. Scale bars: 20 μm.

After IIL were cultured with a Caco-2 cell monolayer in semisolid MEM for 2 h, IIL invasion of the monolayer was examined by microscopy. The non-invaded larvae were coiled on the surface of the monolayer (Figure 2A), the invaded larvae migrated within the cell monolayer and left a migratory path in the monolayer, and dead cells remained (Figures 2B and C). The cell nuclei were dyed orange with PI, the ESPs secreted by the IIL were recognized with anti-ESP immune serum, and green fluorescence staining in the larval migratory path was observed (Figure 2D). Following agarose removal, the nuclei of damaged cells were intensely and uniformly stained orange by PI, showing the serpentine trail followed by the parasite (Figure 2E), and the IIL ESPs within the migratory path were probed using anti-ESP immune serum (Figure 2F).

**Figure 3:**
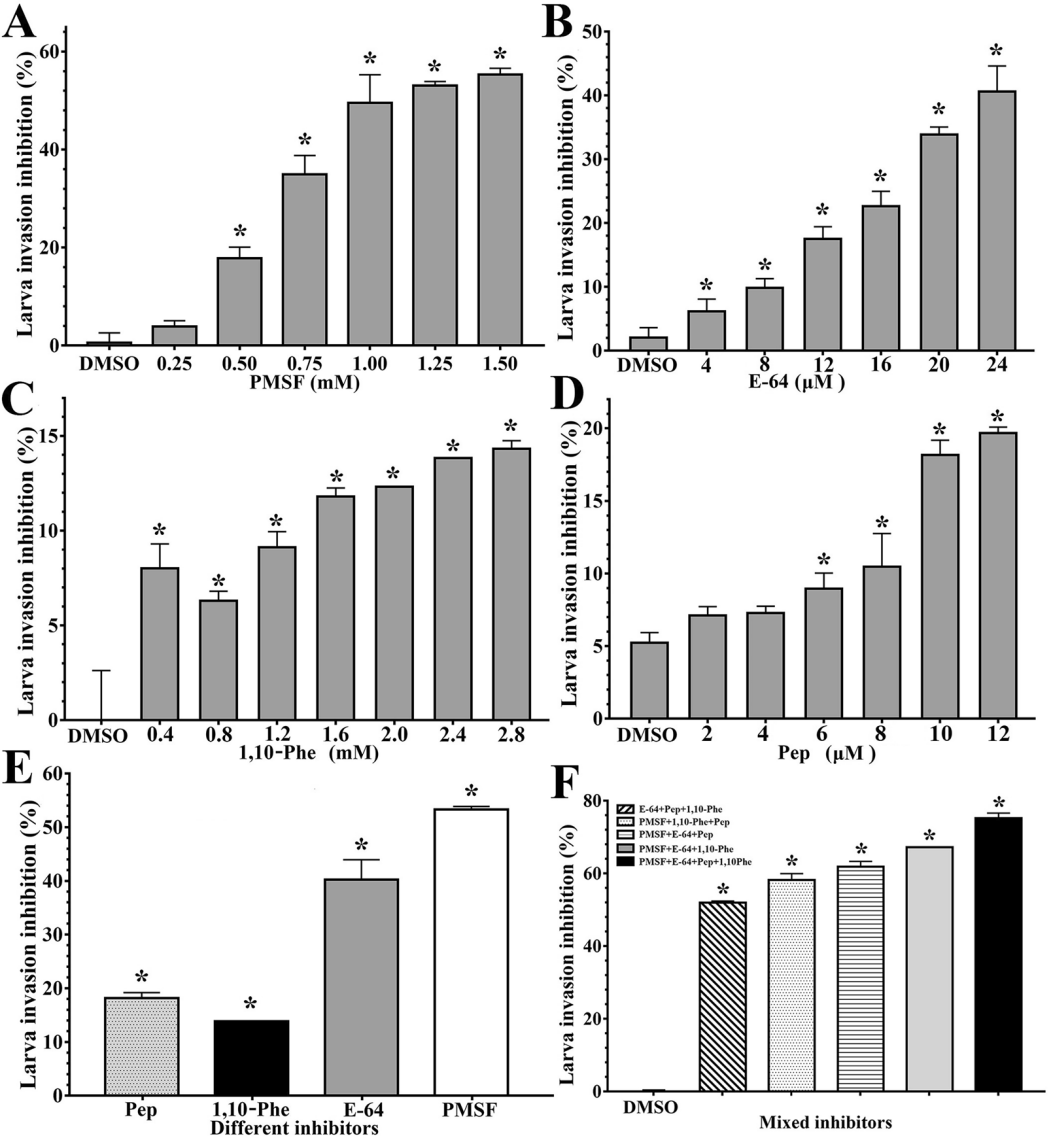
**Inhibition of larval invasion of a Caco-2 cell monolayer with different concentrations of PMSF, E-64, 1,10-Phe or pepstatin**. Various concentrations of diverse enzyme inhibitors were replenished in semisolid MEM, which was used to co-culture IIL with a Caco-2 cell monolayer for 2 h, and worm intrusion into the Caco-2 cell monolayer was examined under a microscope. The inhibitory effects of the various inhibitors on larval invasion are expressed as the inhibition (%) normalized to the PBS control group, and the results are expressed as the mean ± SD of three independent tests. **A** Various concentrations of PMSF. **B** Various concentrations of E-64. **C** Various concentrations of 1,10-phenanthroline (1,10-Phe). **D **Various concentrations of pepstatin. **E** Comparison of the suppressive effects of the 4 kinds of inhibitors on IIL invasion of Caco-2 cell monolayers when the optimal inhibitor concentrations (1.25 mM PMSF, 24 μM E-64, 2.4 mM 1,10-Phe and 10 μM pepstatin) were used. **F:** Comparison of the suppressive effects of mixtures of the various inhibitors on IIL invasion of Caco-2 cell monolayers. *P < 0.0001 compared with the DMSO control group.

After different concentrations (0.25–1.50 mM) of PMSF were added to the semisolid medium and cultured with IIL for 2 h, compared with PBS control treatment, the percent inhibition values for IIL invasion in the monolayer were 3.85, 17.79, 34.97, 49.53, 53.28 and 55.31%, respectively (*F* = 189.030, *P* < 0.0001), and the inhibitory effect was dependent on the PMSF concentration (*r* = 0.972, *P* < 0.0001) (Figure 3A). The rates of IIL invasion inhibition achieved with various E-64 concentrations (4–24 μM) were 6.15, 9.85, 17.52, 22.65, 33.86 and 40.59%, respectively (*F* = 122.878, *P* < 0.0001), and the inhibitory effect was correlated with the E-64 concentration (*r* = 0.991, *P* < 0.0001) (Figure 3B). The inhibition of larval invasion by various 1,10-Phe concentrations (0.4–2.8 mM) was significant (*F* = 53.762, *P* < 0.0001), and the suppressive efficacy of 1,10-Phe was dose dependent (Figure 3C). When different doses (2–12 μM) of pepstatin were used, larval penetration was inhibited by 7.10, 7.28, 8.96, 10.46, 18.17 and 19.68%, respectively (*F* = 62.864, *P* < 0.0001) (Figure 3D). When the suppressive effects of the 4 inhibitors achieved with their optimal concentrations (1.25 mM PMSF, 24 μM E-64, 2.4 mM 1,10-Phe and 10 μM pepstatin) were compared, the rates of IIL invasion inhibition by PMSF, E-64, 1,10-Phe and pepstatin were 53.28, 40.59, 13.83 and 18.17%, respectively (*F* = 238.988, *P* < 0.0001) (Figure 3E). When mixtures of various inhibitors (PMSF + E-64 + 1,10-Phe + pepstatin, PMSF + E-64 + 1,10-Phe, PMSF + E-64 + pepstatin, PMSF + 1,10-Phe + pepstatin, and E-64 + 1,10-Phe + pepstatin) were used, the rates of IIL invasion inhibition by the 5 inhibitor mixtures were 75.18, 67.18, 61.83, 57.00 and 51.95%, respectively (*F* = 2783.849, *P* < 0.0001) (Figure 3F). The results indicated that PMSF had the strongest inhibitory effect on IIL invasion, followed by E-64, 1,10-Phe and pepstatin, suggesting that the IIL invasion-related proteases are principally serine proteases and cysteine proteases but that aspartic proteases and metalloproteases are also involved in IIL invasion of the gut epithelium.

### Gut epithelial TJ protein disruption caused by various proteases in IIL ESPs

**Figure 4:**
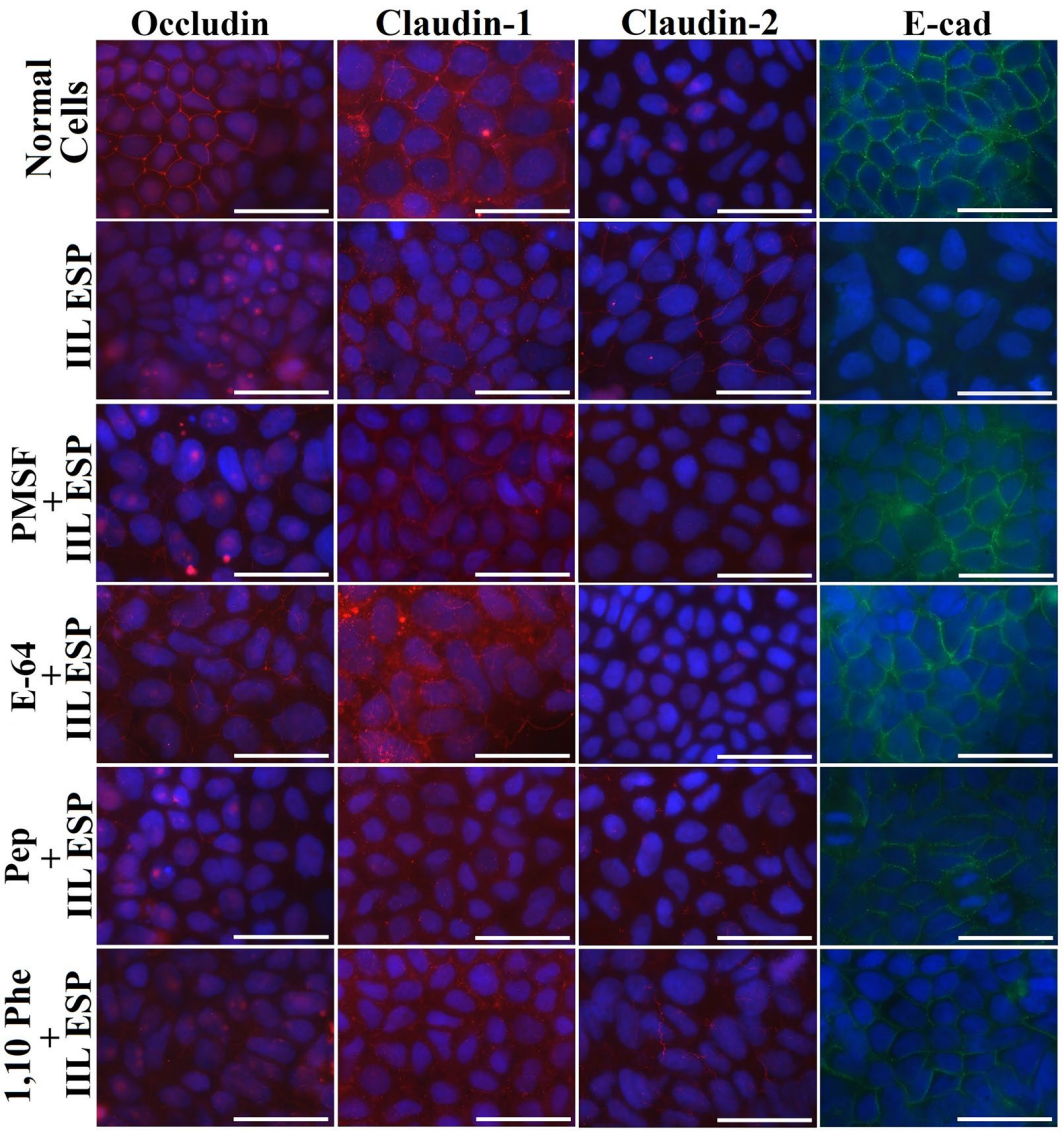
**Immunofluorescence staining to localize tight and adherens junction proteins after Caco-2 cell monolayers were incubated with IIL ESPs treated with various single inhibitors.** Caco-2 cell monolayers were incubated at 37 ℃ for 2 h with IIL ESPs pre-treated using various individual inhibitors (1.25 mM PMSF, 24 μM E-64, 2.4 mM 1,10-Phe or 10 μM pepstatin). Normal Caco-2 cells treated with 0.1% DMSO were used as a negative control, and 20 μg/mL ESPs without inhibitors was used to assess the hydrolysis of TJ proteins. The cells were fixed with 4% paraformaldehyde, permeabilized for 10 min with 0.25% Triton X-100 and blocked with 1% BSA. The cells were probed with primary antibodies against occludin, claudin-1, claudin-2 or E-cad and then incubated with FITC- or Cy3-conjugated secondary antibodies. Cell nuclei were stained blue with DAPI before being observed under a fluorescence microscope (1000×). Scale bars: 50 μm.

**Figure 5:**
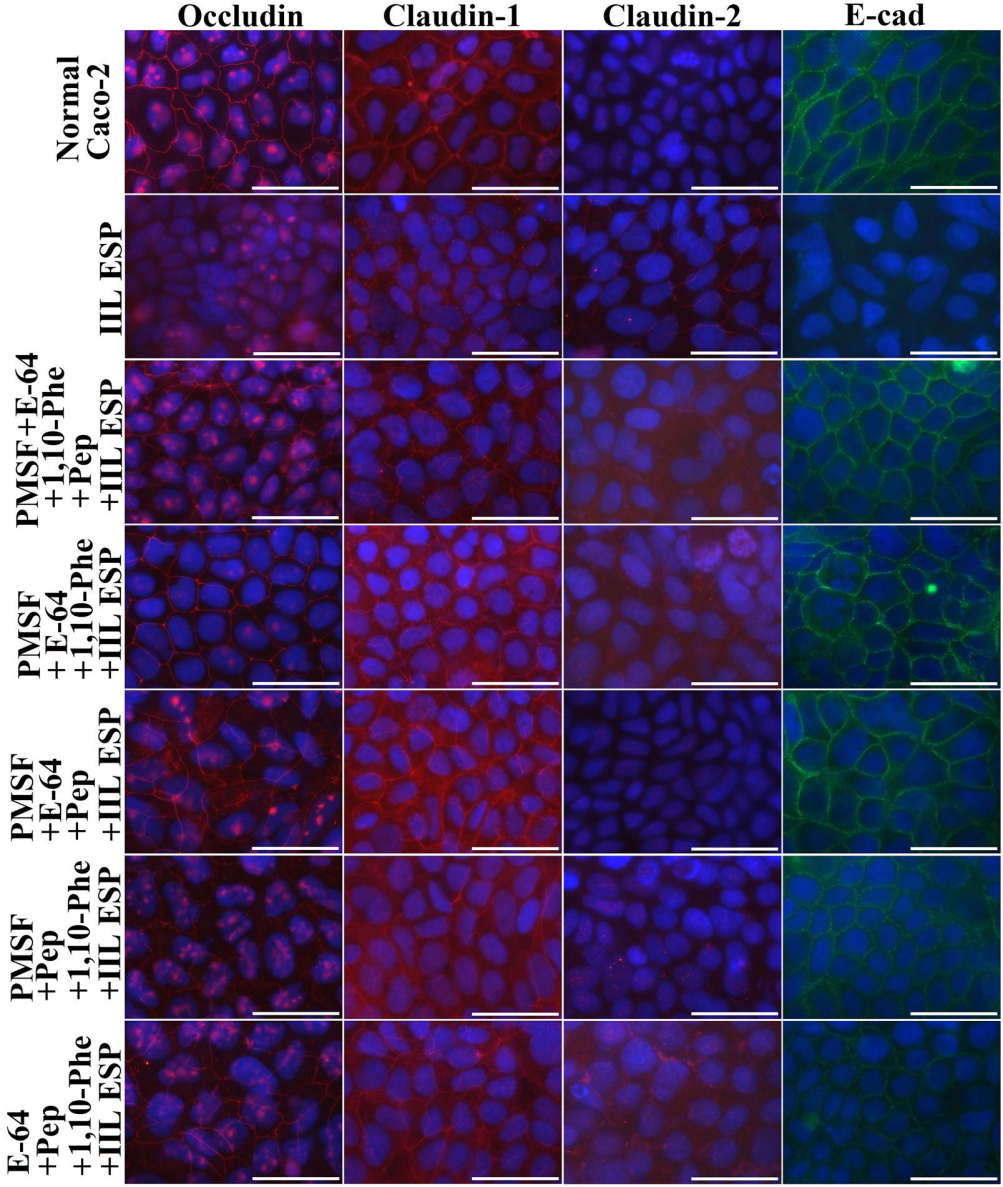
**Immunofluorescence staining to localize tight and adherens junction proteins after Caco-2 cells were incubated with IIL ESPs treated with various inhibitor mixtures.** IIL ESPs pre-incubated with different mixtures of 4 inhibitors were added to Caco-2 cell monolayers and incubated at 37 °C for 2 h. The cells were examined under a fluorescence microscope, and the cell nuclei were stained blue with DAPI. When the IIL ESPs were pre-treated with the 4-inhibitor mixtures (PMSF + E-64 + 1,10-Phe + pepstatin), the degradation of occludin, claudin-1 and E-cad by the IIL ESPs was completely blocked, while claudin-2 expression was restored. Scale bars: 50 μm.

To identify the main proteases in IIL ESPs related to gut barrier disruption, the damage caused by IIL ESPs to epithelial monolayers was investigated. The distribution and integrity of tight (occludin, claudin-1, and claudin-2) and adherens (E-cad) junction proteins were examined under a fluorescence microscope upon the addition of ESPs (20 μg/mL) to Caco-2 cell monolayers. In normal Caco-2 cells, occludin, claudin-1 and E-cad were distributed around the cell border; there was a small quantity of claudin-2 proteins around the cells (Figure 4). Moreover, occludin was also scattered within cell nuclei (Figure 5). However, after cells were treated with ESPs, the quantities of occludin, claudin-1 and E-cad around the cells were clearly reduced, and the continuous cell border staining was lost, showing that ESP treatment accelerated the degradation of occludin, claudin-1 and E-cad in epithelial cells, but claudin-2 expression was increased. Moreover, when ESPs treated with PMSF and E-64 were used, the cell border staining for occludin, claudin-1 and E-cad was more evident than that in the ESP, ESP + 1,10-Phe and ESP + pepstatin groups but was still weaker than that in the normal cell group, and claudin-2 expression was inhibited (Figure 4), indicating that the epithelial monolayer damage caused by IIL ESPs was blocked by pre-incubation with the inhibitors PMSF and E-64. The results suggested that occludin, claudin-1 and E-cad were degraded mainly by serine proteases and cysteine proteases in IIL ESPs, whereas these two types of proteases stimulated and upregulated claudin-2 expression.

When IIL ESPs pre-incubated with different mixtures of the 4 inhibitors (PMSF, E-64, 1,10-Phe and pepstatin) were added to Caco-2 cell monolayers, the mixtures of the 4 inhibitors completely inhibited the degradation of occludin, claudin-1 and E-cad by the ESPs and restored claudin-2 expression (Figure 5). When mixtures of 3 inhibitors were used, the suppression of the hydrolysis of occludin, claudin-1 and E-cad was similar to that achieved with the mixtures of the 4 inhibitors only if PMSF and E-64 were included, further demonstrating that the degradation of occludin, claudin-1 and E-cad and damage to gut epithelial integrity in the cell monolayer were mainly caused by serine proteases and cysteine proteases in the ESPs. The results suggested that IIL ESPs have the capacity to destroy the gut epithelial barrier and mediate larval invasion of the gut mucosa.

### Western blot analysis of TJ protein damage caused by various proteases in IIL ESPs

**Figure 6:**
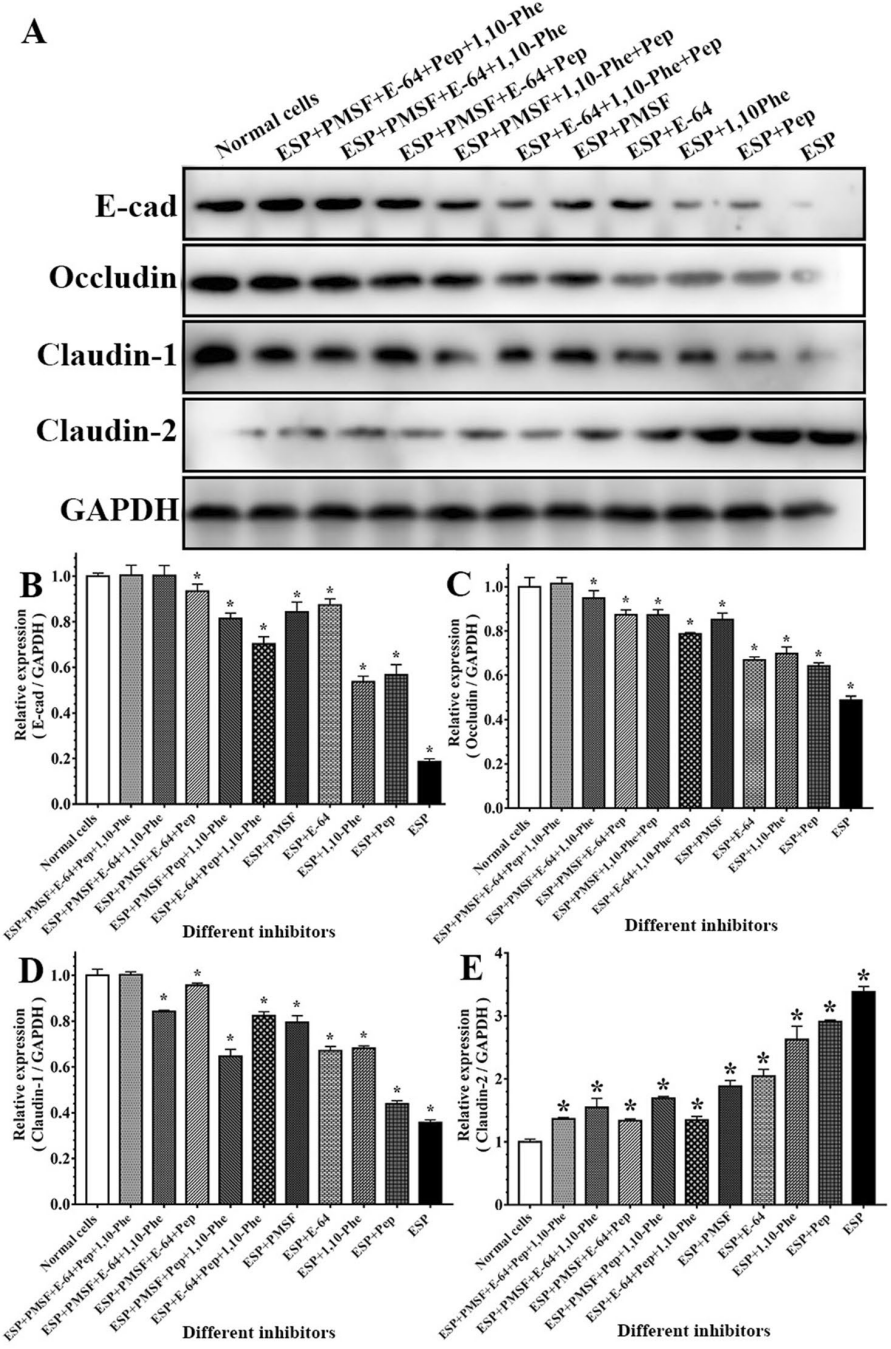
**Western blot analysis of the hydrolysis of TJ and adherens proteins in Caco-2 cells by various proteases in IIL ESPs. A** Caco-2 cell monolayers were incubated with IIL ESPs pre-treated with various inhibitors (1.25 mM PMSF, 24 μM E-64, 2.4 mM 1,10-Phe or 10 μM pepstatin) alone or in mixtures at 37 ℃ for 2 h. Normal Caco-2 cells treated with 0.1% DMSO were used as a negative control, and 20 μg/mL ESP without inhibitors was used to assess the hydrolysis of TJ proteins. The expression of TJ and adherens junction proteins was analysed by Western blotting. GAPDH was used as an internal reference control. **B–E** Densitometric analysis of the bands obtained in **A** for E-cad (**B**), occludin (**C**), claudin-1 (**D**) and claudin-2 (**E**) relative to the GAPDH band. *P < 0.05 compared to normal Caco-2 cells.

Caco-2 cell monolayers were incubated with IIL ESPs pre-treated with various inhibitors at 37 ℃ for 2 h. Western blot results showed that different inhibitors had various suppressive effects on the hydrolysis of Caco-2 cell tight and adherens junction proteins by the IIL ESPs. Compared to those in normal Caco-2 cells, the expression levels of E-cad, occludin and claudin-1 in Caco-2 cells incubated with IIL ESPs were obviously decreased (*F* = 5589.569, *F* = 366.977, *F* = 1428.259, *P* < 0.0001), but the expression levels of claudin-2 were evidently increased (*F* = 1817.892, *P* < 0.0001) (Figure 6). However, when mixtures of the 4 inhibitors were used, the expression levels of E-cad, occludin and claudin-1 were upregulated to the expression levels in normal Caco-2 cells (*F* = 0.022, *F* = 0.224, *F* = 0.024, *P* > 0.05). When PMSF, E-64 or various 3-inhibitor mixtures containing PMSF and E-64 were used, the suppression of the hydrolysis of occludin, claudin-1 and E-cad was more remarkable than that achieved with pepstatin, 1,10-Phe or the other 3-inhibitor mixtures lacking PMSF and E-64 (*F* = 68.419, *F* = 206.189, *F* = 68.594, *P* < 0.0001). Moreover, after PMSF, E-64 or various 3-inhibitor mixtures containing PMSF and E-64 were used, the protein expression levels of claudin-2 were reduced compared to those in the IIL ESP, pepstatin and 1,10-Phe groups (*F* = 88.046, *P* < 0.0001). The results further indicated that the TJ proteins (occludin, claudin-1 and E-cad) of the gut epithelium were principally hydrolysed by serine proteases and cysteine proteases in IIL ESPs and that these two types of proteases also upregulated claudin-2 expression in the gut epithelium.

### Gut epithelial TJ protein disruption by various proteases in IIL ESPs measured with Transwell assays

**Figure 7:**
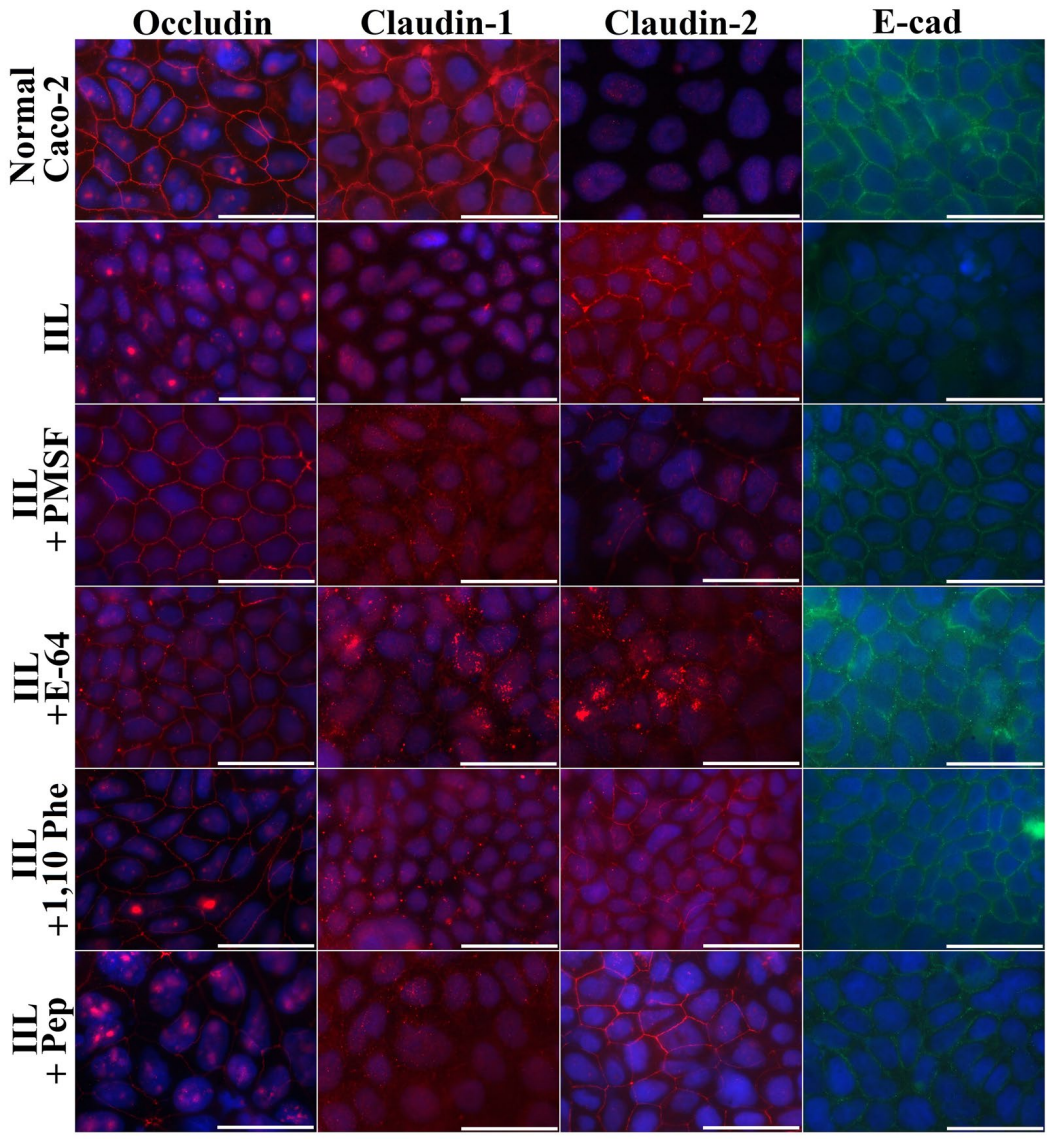
**Immunofluorescence staining to localize TJ and adherens junction proteins after Caco-2 cells were incubated with IIL ESPs treated with various inhibitors in a Transwell system.** Caco-2 cells were cultured on glass coverslips in a Transwell system, and 200 IIL pre-treated with various inhibitors (1.25 mM PMSF, 24 μM E-64, 2.4 mM 1,10-Phe or 10 μM pepstatin) were incubated on the insert at 37 ℃ for 2 h. Normal Caco-2 cells treated with 0.1% DMSO were used as a negative control, and normal IIL not treated with inhibitors were used to assess the ESP-mediated -hydrolysis of TJ proteins. The cells were fixed, blocked, and probed with antibodies against occludin, claudin-1, claudin-2 or E-cad followed by FITC- or Cy3-conjugated secondary antibodies. Cell nuclei were dyed blue with DAPI before examination by fluorescence microscopy (1000×). Scale bars: 50 μm.

**Figure 8:**
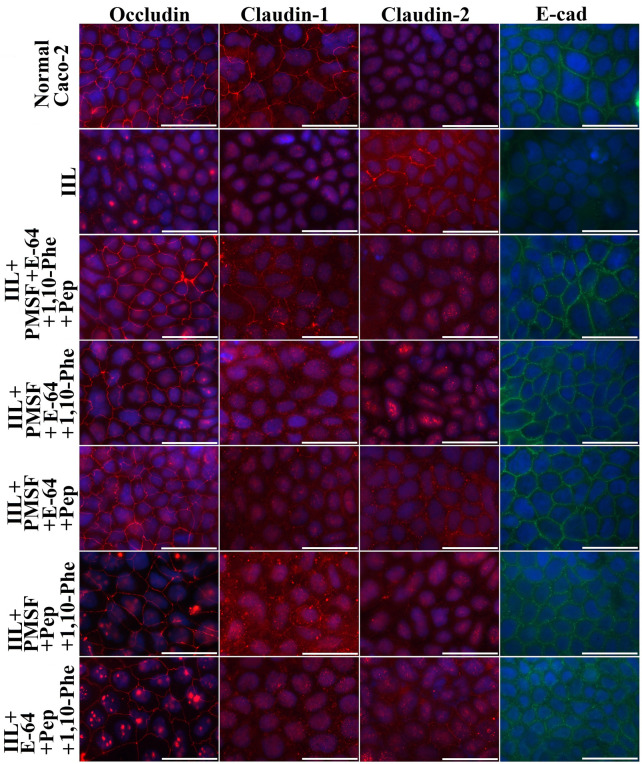
**Immunofluorescence localization of TJ and adherens junction proteins after Caco-2 cells were incubated with IIL ESPs treated with inhibitor mixtures in a Transwell system.** Caco-2 cells were cultured on glass coverslips in a Transwell system, and 200 IIL pre-treated with various inhibitor mixtures were incubated on the insert at 37 ℃ for 2 h. The cells were fixed, blocked, probed with antibodies against occludin, claudin-1, claudin-2 or E-cad and then stained using FITC- or Cy3-labelled secondary antibodies. Cell nuclei were stained blue with DAPI before examination by fluorescence microscopy (1000×). Scale bars: 50 μm.

The results of a Transwell assay showed that the direct disruption of TJ proteins by IIL ESPs was similar to that achieved with ESPs collected from in vitro-cultured IIL. PMSF and E-64 had obvious inhibitory effects on the IIL ESP-mediated hydrolysis of occludin, claudin-1 and E-cad, and the inhibitors also suppressed the expression-enhancing effect of IIL ESPs on claudin-2 (Figure 7). When IIL were treated with various inhibitor mixtures, the fluorescence intensities of occludin, claudin-1 and E-cad were obviously decreased in the E-64 + 1,10-Phe + pepstatin and PMSF + 1,10-Phe + pepstatin groups compared with the other three groups (PMSF + E-64 + 1,10-Phe, PMSF + E-64 + pepstatin, and PMSF + E-64 + 1,10-Phe + pepstatin). However, claudin-2 staining was increased compared to the staining for the other TJ proteins (Figure 8). The results also demonstrated that the degradation of occludin, claudin-1 and E-cad in cell monolayers was principally mediated by serine proteases and cysteine proteases in IIL ESPs, whereas IIL ESPs increased claudin-2 expression.

### Inhibition of the ESP-mediated hydrolysis of collagen I and fibronectin by various inhibitors

**Figure 9:**
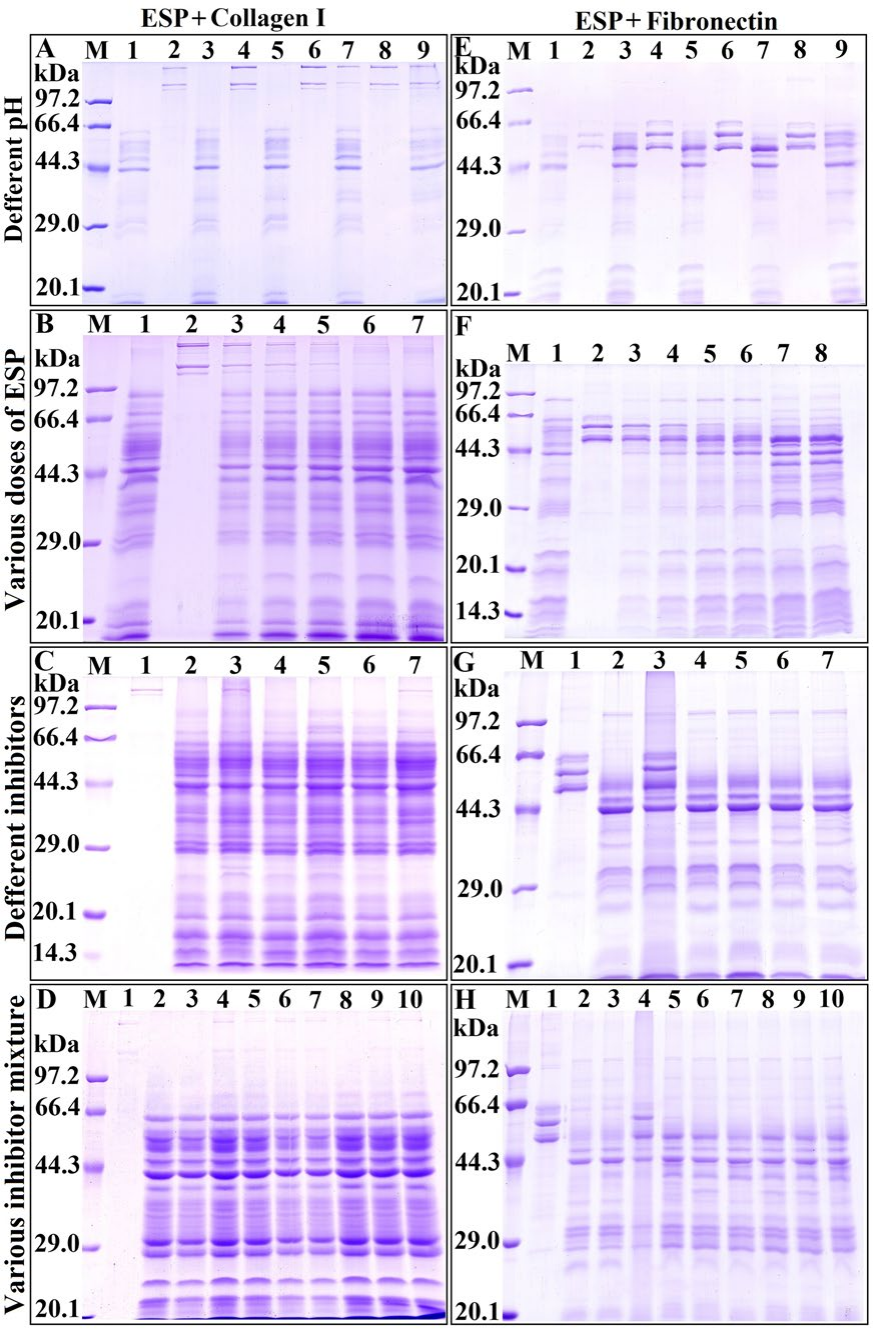
**SDS–PAGE analyses of the main proteases in ESPs capable of hydrolysing collagen I (COI) and fibronectin (Fib).** Lane M: protein marker. **A** ESPs (8.25 μg) hydrolysed COI (4.5 µg) at different pH values. Lane 1: ESPs; Lanes 2, 4, 6 and 8: COI at pH 4.5, 5.5, 6.5 and 7.5, respectively; Lanes 3, 5, 7 and 9: ESPs + COI at pH 4.5, 5.5, 6.5 and 7.5, respectively. **B** Various ESP doses degraded COI (4.5 µg) at pH 5.5. Lane 1: ESPs; Lane 2: COI; Lanes 3–7: ESPs (5.50, 8.25, 11.00, 13.75 and 16.50 μg, respectively) + COI. **C** Various inhibitors (1 mM PMSF, 10 mM EDTA, 20 μM E-64 or 10 μM pepstatin) suppressed the ESP-mediated hydrolysis of COI. Lane 1: COI; Lane 2: ESPs + COI; Lane 3: inactivated ESPs + COI; Lane 4: (ESPs + E-64) + COI; Lane 5: (ESPs + PMSF) + COI; Lane 6: (ESPs + pepstatin) + COI; Lane 7: (ESPs + EDTA) + COI. **D** Inhibitor mixtures suppressed the ESP-mediated hydrolysis of COI. Lane 1: COI; Lane 2: ESPs; Lane 3: ESPs + COI; Lane 4: inactivated ESPs + COI; Lane 5: (ESPs + E-64 + PMSF + pepstatin) + COI; Lane 6: (ESPs + E-64 + PMSF + EDTA) + COI; Lane 7: (ESPs + E-64 + pepstatin + EDTA) + COI; Lane 8: (ESPs + PMSF + pepstatin + EDTA) + COI; Lane 9: (ESPs + E-64 + PMSF + pepstatin + EDTA) + COI; Lane 10: (ESPs + inhibitor cocktail) + COI. **E** ESPs (8.25 μg) hydrolysed Fib (5 μg) at various pH values. Lane 1: ESPs; Lanes 2, 4, 6 and 8: Fib; Lanes 3, 5, 7, and 9: ESPs + Fib at pH 4.5, 5.5, 6.5 and 7.5, respectively. **F** Various ESP doses hydrolysed Fib. Lane 1: ESPs; Lane 2: Fib; Lanes 3–8: ESPs (2.5, 5.0, 7.5, 10.0, 12.5 and 15.0 μg, respectively) + Fib. **G** Inhibitors suppressed the ESP-mediated hydrolysis of Fib. Lane 1: Fib; Lane 2: ESPs + Fib; Lane 3: inactivated ESPs + Fib; Lane 4: (ESPs + E-64) + Fib; Lane 5: (ESPs + PMSF) + Fib; Lane 6: (ESPs + pepstatin) + Fib; Lane 7: (ESPs + EDTA) + Fib. **H** Inhibitor mixtures suppressed the ESP-mediated hydrolysis of Fib. Lane 1: Fib; Lane 2: ESPs; Lane 3: ESPs + Fib; Lane 4: inactivated ESPs + Fib; Lane 5: (ESPs + E-64 + PMSF + pepstatin) + Fib; Lane 6: (ESPs + E-64 + PMSF + EDTA) + Fib; Lane 7: (ESPs + E-64 + pepstatin + EDTA) + Fib; Lane 8: (ESPs + PMSF + pepstatin + EDTA) + Fib; Lane 9: (ESPs + E-64 + PMSF + pepstatin + EDTA) + Fib; Lane 10: (ESPs + inhibitor cocktail) + Fib.

SDS–PAGE analysis revealed that the optimum pH value for the ESP-mediated hydrolysis of collagen I was pH 5.5 (Figure 9A). Different ESP doses (5.50, 8.25, 11.00, 13.75, and 16.50 μg) could degrade 4.5 μg of collagen I (Figure 9B), and the ESP-mediated hydrolysis of collagen I was blocked by EDTA (Figure 9C). Various inhibitor mixtures containing EDTA significantly suppressed the ESP-mediated hydrolysis of collagen I, but a mixture (E-64 + PMSF + pepstatin) without EDTA did not inhibit the hydrolysis of collagen I (Figure 9D). The results suggested that the ESP proteases involved in hydrolysing collagen I were mainly metalloproteases.

When fibronectin was used as the substrate, the optimal pH for ESP hydrolytic activity was pH 6.5 (Figure 9E). The optimum dose of ESPs for hydrolysis of 5 µg of Fib was 12.5 µg (Figure 9F). None of the 4 protease inhibitors (E-64, PMSF, pepstatin or EDTA) inhibited the ESP-mediated hydrolysis of fibronectin (Figure 9G). Diverse inhibitor mixtures containing PMSF suppressed the ESP-mediated hydrolysis of fibronectin, but a mixture without PMSF did not inhibit fibronectin hydrolysis (Figure 9H). The results suggested that the ESP proteases involved in hydrolysing fibronectin were primarily serine proteases.

### Protease inhibitors impaired larval infectivity and survival in the host

**Figure 10:**
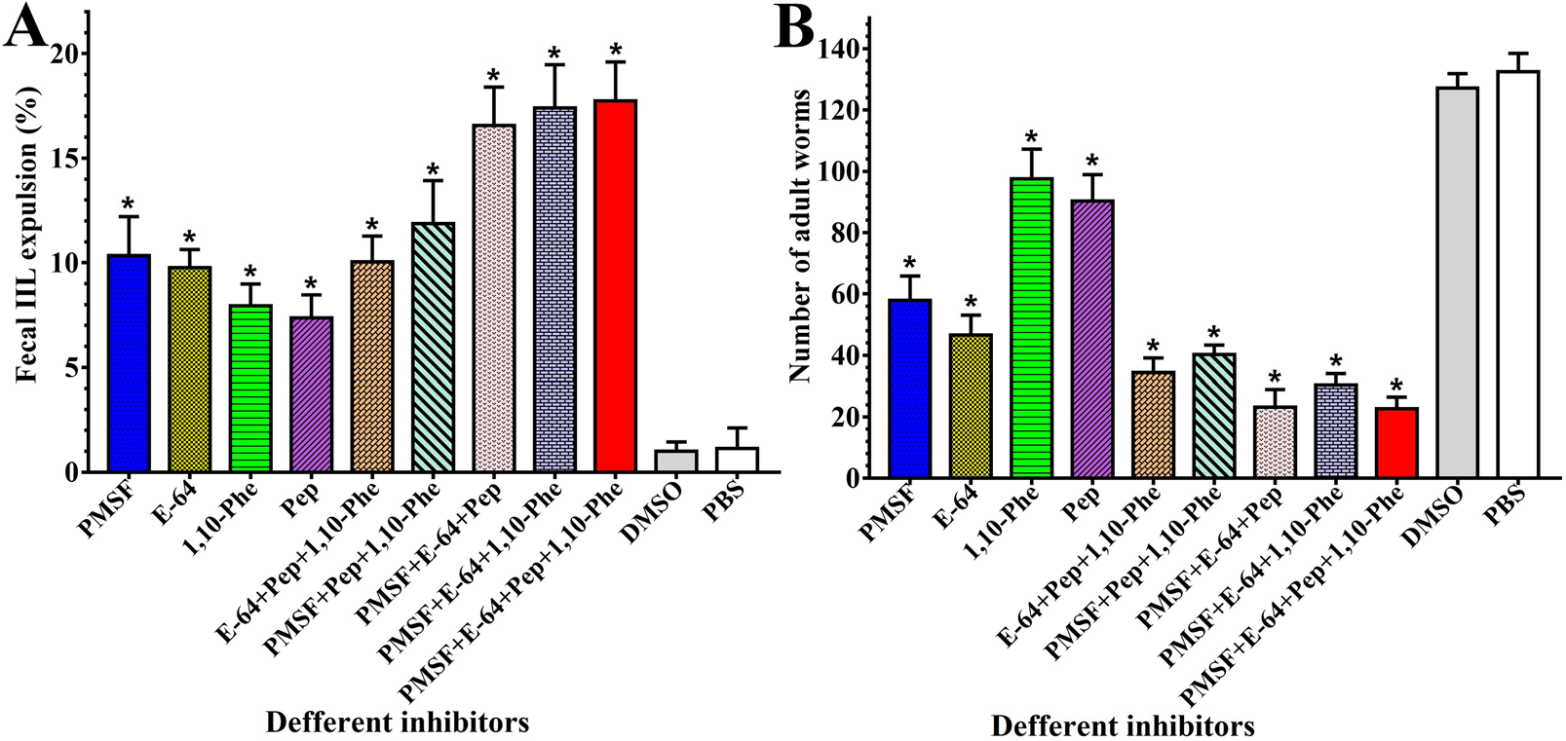
**The IIL expelled in faeces and intestinal adult worms in mice infected with larvae treated with various inhibitors. A** Expelled IIL larvae in faeces within 12 h following challenge infection (*n* = 10). **B** Intestinal adult worm burden (*n* = 10). The data are shown as the mean ± SD of the various inhibitor, DMSO control and PBS control groups. ML treated with 0.1% DMSO or PBS were used as the negative or normal control. *P < 0.0001 relative to the PBS control group.

After treatment with various inhibitors, the IIL expelled from the gut within 12 h after challenge were recovered. The results showed that the IIL expulsion rates of mice infected with ML treated with various inhibitors (PMSF, E-64, 1,10-Phe, or pepstatin) alone or in mixtures were significantly higher than that of mice in the PBS group (*F* = 89.311, *P* < 0.0001), but the solvent DMSO control group did not exhibit any evident worm expulsion compared to the PBS group (*P* > 0.05) (Figure 10A). Furthermore, when a mixture of the 4 inhibitors and other various inhibitor mixtures containing PMSF and E-64 were used, the IIL expulsion rates were obviously higher than those of the individual inhibitors and inhibitor mixtures without PMSF and E-64 (*F* = 41.597, *F* = 19.323, *P* < 0.0001). Compared to those in the PBS group, the mice which were inoculated with larvae treated with PMSF, E-64, 1,10-Phe or pepstatin showed 56.30, 64.91, 26.42 and 31.85% reductions in gut adult worms at 6 days after challenge (*F* = 188.163, *P* < 0.0001). The adult worm burdens of the PMSF and E-64 groups were remarkably lower than those of the 1,10-Phe and pepstatin control group (*F* = 107.484, *F* = 86.087, *P* < 0.0001). Moreover, the adult worm burdens of various mixed inhibitor groups were distinctly inferior to those of the 1,10-Phe and pepstatin control group (*F* = 258.082, *F* = 233.951, *P* < 0.0001) (Figure 10B). The results demonstrated that the protease inhibitors PMSF and E-64 prominently reduced larval infectivity and impeded larval development and survival in the host intestines, suggesting that serine proteases and cysteine proteases play vital roles in *T. spiralis* larval invasion, growth and survival, while other proteases (aspartic proteases and metalloproteases) also participate in larval invasion and development.

## Discussion

The mucosal surfaces of the intestinal tract are the most important site for intestinal parasite entry into the host and are important sites of parasitic diseases. An essential function of the intestinal epithelium is to act as a natural barrier that limits pathogen invasion. IECs, which line the gut mucosal surfaces, are an important mechanical barrier that prevent parasite invasion of host tissues, and they are also the first site of interaction between the parasites and host. Additionally, IECs perform an important function in ion transport and fluid absorption. Gut epithelial barrier function requires a continuous cell layer as well as the junctions that seal the paracellular space between IECs. TJs are intercellular junctions between IECs, which are essential for gut epithelial adhesion and barrier function. This barrier regulates nutrient and water absorption while limiting the invasion of bacteria and other pathogens into the bowel lumen [53]. In the IEC monolayer, there is an apical junction complex (AJC) composed of TJs, adherens junctions and desmosomes. AJCs between cells form a sealed barrier to prevent microbes and antigens from moving between epithelial cells. TJ proteins include many transmembrane proteins. Mammalian TJ proteins are mainly composed of claudin 1-claudin 27, occludin and junction adhesion molecules (JAMs) [54]. The presence of claudins in TJs is considered to determine the “tightness” of the specific epithelium, and adherens junction proteins are mainly cadherin and connective protein. Disruption of TJs can result in gut epithelial barrier damage and facilitate mucosal invasion by *Entamoeba histolytica* trophozoites [55]. The ESPs of *Haemonchus contortus* and *Teladorsagia circumcincta* adults disrupt the TJs of cultured epithelial cells. Gut epithelial permeability is notably elevated, and the degradation of the TJ protein occludin also occurs during *T. spiralis* infection [56]. During *T. spiralis* infection, the expression of the gut epithelial TJ proteins occludin and claudin-3 was found to be downregulated beginning at 2 days post-infection, whereas claudin-2 was overexpressed. These changes were associated with increases in in vitro and in vivo epithelial permeability [57].

Proteases play key roles during physiological processes of the gut epithelial barrier, and they are divided into four different kinds, namely, serine proteases, cysteine proteases, aspartic proteases and metalloproteinases [58]. Proteases have received notable attention as important biological molecules in the lifecycle of parasites. Gut proteases produced by intestinal parasites have multiple effects on intestinal barrier function mediated by acting on several elements forming intercellular tight junctions between IECs [18]. They are involved in the processes of host tissue invasion and degradation, larval migration, nutrition, and host immune response escape [15, 16]. Various proteases have been identified in the ESPs and crude antigens of *T. spiralis* ML and adult worms by zymography, proteomics and immunoproteomics [10, 11, 59]. As IIL are the first invasive stage of the *T. spiralis* lifecycle, penetrating the host gut mucosa, different proteases produced during the IIL stage were also screened and identified [9, 27]. However, which kinds of IIL proteases play vital roles in IIL penetration of the gut epithelium is not clear. The proteases in the ESPs produced by *T. spiralis* IIL are first exposed directly to the host gut intestinal mucosa; they might hydrolyse the TJ proteins of IECs, destroy the barrier integrity of the intestinal epithelium, and mediate IIL invasion of the gut mucosa [60]. Therefore, in the present study, the role of IIL ESPs in host gut epithelium invasion was first investigated through an in vitro model of IIL invasion of IEC monolayers, and then which kinds of main proteases are involved in the destruction and invasion of the intestinal epithelium were ascertained by using various enzyme inhibitors and an animal infection experiment.

As shown in Figure 2, IIL invaded and migrated within Caco-2 cell monolayers, leaving a serpentine migratory trail in each monolayer. The migratory paths were probed using anti-ESP immune serum, which suggested that the IIL produced and left ESPs during larval intrusion and migration. When four kinds of inhibitors (PMSF, E-64, 1,10-Phe and pepstatin) were added to the co-culture medium for IIL and Caco-2 cells for 2 h, all four inhibitors suppressed larval invasion of the monolayer, and the suppressive effects were dose dependent. The results also indicated that PMSF had the strongest inhibitory effect on larval invasion, followed by E-64, 1,10-Phe and pepstatin, suggesting that the IIL invasion-related proteases are principally serine proteases and cysteine proteases but that aspartic proteases and metalloproteases are also involved in IIL invasion of the gut epithelium. Previous studies have shown that recombinant serine proteases [61–63], cysteine proteases [17, 41, 64], aspartic proteases [23] and metalloproteases [33, 52] from *T. spiralis* larvae promote larval invasion of IECs and the gut mucosa, whereas specific antibodies and siRNAs specific for these recombinant proteases impede penetration of the gut epithelium. The results suggested the important functions of the IIL ESP proteases in larval invasion of the IEC monolayer.

The IIFT results showed that the gut epithelial TJ proteins occludin, claudin-1 and E-cad were visible around the borders of normal Caco-2 cells, while only a small quantity of claudin-2 was present. After ESP treatment, the quantities of occludin, claudin-1 and E-cad around the cells were remarkably reduced, as they lost their continuous cell border staining, but claudin-2 expression was increased, suggesting that the ESPs degraded occludin, claudin-1 and E-cad but stimulated claudin-2 expression. When IIL ESPs treated with PMSF and E-64 were used, the cell border staining of occludin, claudin-1 and E-cad was more evident than that in the ESP, ESP + 1,10-Phe and ESP + pepstatin groups but was still weaker than that in the normal cell group, and claudin-2 expression was inhibited. When different mixtures of the 4 inhibitors (PMSF, E-64, 1,10-Phe and pepstatin) were used, the mixtures containing all 4 inhibitors completely blocked the degradation of occludin, claudin-1 and E-cad by ESPs and restored claudin-2 expression. When different mixtures of 3 inhibitors were used, the suppression of the hydrolysis of occludin, claudin-1 and E-cad was similar to that achieved with the mixtures of 4 inhibitors only when PMSF and E-64 were included in the mixture. The results suggested that occludin, claudin-1 and E-cad were degraded mainly by serine proteases and cysteine proteases in ESPs, whereas these two types of proteases induced upregulated claudin-2 expression, demonstrating that disruption of gut epithelial integrity and damage to the gut epithelium were mainly caused by serine proteases and cysteine proteases in IIL ESPs, which might destroy the natural gut epithelial barrier and facilitate larval invasion of the gut mucosa. As shown in Figures 7 and 8, when the Transwell assay was performed, the direct disruption of gut epithelial integrity by IIL ESPs was similar to that observed with ESPs added directly to a Caco-2 cell monolayer. The Transwell assay suggests that the degradation of the intestinal epithelial barrier does not require direct contact between IIL and gut epithelial cells; instead, it is the consequence of the action of IIL ESPs. The Western blot results showed that ESPs clearly hydrolysed occludin, claudin-1 and E-cad and increased claudin-2. The hydrolysis of occludin, claudin-1 and E-cad in the gut epithelium was also principally mediated by serine proteases and cysteine proteases in ESPs. Previous studies showed that giardipain-1 (a mature cathepsin B-like enzyme) from *Giardia lamblia* trophozoites degraded the tight junction proteins occludin and claudin-1 in a rat intestinal epithelial cell (IEC6) monolayer, that the epithelial monolayer damage caused by giardipain-1 was blocked by preincubation with the inhibitor E-64 and that silencing the giardipain-1 gene in trophozoites lowered the proteolytic activity of giardipain-1 and reduced the damage to cell monolayers [19]. Cysteine proteases secreted by *G. lamblia* disrupt intestinal epithelial cell junctional complexes [29]. These results indicate that cysteine proteases are the main virulence factors of *Giardia* trophozoites. The recombinant *Entamoeba histolytica* cysteine protease (rEhCP112) results in in vitro and in vivo damage to TJ functions, and rEhCP112 degrades claudin-1, thus affecting interepithelial adhesion. EhCP112 could be a potential therapeutic target in amoebiasis [38]. The serine proteases of *T. spiralis* muscle larvae might reduce the expression of TJ proteins via the P38-MAPK signalling pathway and reduce intestinal barrier integrity [65]. The results of this study show that serine proteases and cysteine proteases in ESPs are the main invasive factors of *T. spiralis* IIL that degrade the TJ proteins of the gut epithelium and mediate larval invasion [37, 44, 66].

Collagen is an important component of the basement membrane, which forms the skeleton of the extracellular matrix. Fibronectin is a component of the extracellular matrix that binds to cells. Tight junctions are the structural connections formed between cells and the extracellular matrix. Collagen, fibronectin and tight junction proteins are important components of the cell-to-cell and cell-to-extracellular matrix networks, which together constitute the cellular barrier system of the intestinal epithelium [67]. To further investigate the IIL EPS-mediated disruption of the cellular barrier function of the intestinal epithelium, the ESP-mediated hydrolysis of collagen I and fibronectin was also evaluated in this study. SDS–PAGE results showed that ESPs could hydrolyse collagen I and fibronectin but that inactivated ESPs did not have a hydrolysing effect on collagen I and fibronectin, suggesting that the proteases in ESPs might act by hydrolysing host proteins. In addition, hydrolysis was blocked by inhibitor mixtures containing EDTA or PMSF. The results demonstrated that the principal proteases in ESPs that degrade collagen I and fibronectin are metalloproteases and serine proteases. Furthermore, as shown in Figure 9A, E, ESPs had high hydrolytic activity resulting in collagen I degradation at pH 5.5 and fibronectin degradation at pH 6.5; these pH values are similar to those of the normal intestinal milieu (pH 6 in the duodenum), suggesting that metalloproteases and serine proteases in ESPs also play a crucial role in the degradation of the extracellular matrix of the gut epithelium. The mouse challenge infection results showed that the gut IIL expulsion at 12 hpi of mice infected with ML treated with various single inhibitors (PMSF, E-64, 1,10-Phe, or pepstatin) or mixtures of the inhibitors was significantly higher than that of mice in the PBS group. When various inhibitor mixtures containing PMSF and E-64 were used, IIL expulsion from the gut was obviously higher than that observed with individual inhibitors and inhibitor mixtures not containing PMSF and E-64. At 6 dpi, the mice which were infected with ML treated with PMSF and E-64 showed 56.30 and 64.91% reductions in intestinal adult worms, which were evidently higher than the 26.42 and 31.85% reductions observed in the 1,10-Phe and pepstatin groups. The results indicated that serine proteases and cysteine proteases play major roles in *T. spiralis* IIL invasion, growth and survival, but other proteases (aspartic proteases and metalloproteases) also participate in larval invasion and development [17, 23, 66]. The results are similar to those of our previous studies. By using zymography combined with shotgun LC–MS/MS, a total of 30 T*. spiralis* proteins were identified in IIL ESPs, and they consisted principally of 19 serine proteases as well as 7 metalloproteases and 3 cysteine proteinases. qPCR confirmed that four proteases (two serine proteases, a cysteine protease and a metalloproteinase) were obviously highly expressed in the IIL stage compared to the ML stage [68]. However, in addition to the direct degradation of gut epithelial TJ proteins by the ESP proteases, the proteases might regulate TJ protein expression (downregulation or overexpression) through different signalling pathways and increase paracellular permeability [65], which needs to be investigated in future experiments. The proteases might act as signalling molecules by activating protease-activated receptor (PAR) family members (PAR 1–4). PAR 2 has been confirmed to be activated by a *T. spiralis* protease and is involved in the regulation of gut inflammation and permeability and ion transport [69]. The results of the animal challenge experiment suggested that serine proteases and cysteine proteases in ESPs also played vital roles in IIL larval invasion, growth and survival in the hosts and that these proteases might be regarded as the main candidate target molecules of vaccines against *T. spiralis* IIL invasion and development.

Additionally, the Caco-2 cell line does not have a mucus layer, and the influence of the mucus layer on IIL ESP-mediated hydrolysis of TJ proteins was not assessed in this study. Intestinal mucus is mainly derived from gut epithelial goblet cells. The mucus layer covers the surface of IECs and plays vital roles in the immune response against intestinal helminth infection and in preventing parasites from penetrating deep tissues such as the IEC monolayer, mucosa and the submucosal layer. The basic component of mucus is mucin, a glycoprotein secreted by goblet cells that aggregates to form a sticky and elastic gelatinous monolayer. A previous study revealed that the ESPs secreted by *Trichuris muris* changed the properties of the mucus barrier, making it more porous by degrading the mucin component of the mucus [18]. The results suggested that *T. spiralis* IIL ESPs might first degrade mucin within the mucus barrier and then pass through the porous mucus layer to disrupt the TJ proteins of the intestinal epithelium. The results of the present study showed that inhibitors of ESP enzymes impeded larval invasion of a cell monolayer, facilitated IIL expulsion from the gut and reduced intestinal adult worm burdens, suggesting that the inhibitors might also interrupt larval biological processes, thereby decreasing larval infectivity, development and survival in hosts [33]. A previous study revealed that 1,10-phenanthroline inhibited the egg production of *Schistosoma mansoni* adult worm pairs in vitro, achieving a 98% egg reduction at 50 µM. When worm pairs were exposed to the inhibitor, the males detached from the dish and released the females, resulting in unpaired worms, which suggested broad and important functions for metalloproteases in *S. mansoni* worms [70].

In conclusion, the results of this study showed that *T. spiralis* IIL ESPs destroyed gut epithelial integrity by degrading the TJ proteins occludin, claudin-1 and E-cad but also stimulated claudin-2 expression. The serine proteases and cysteine proteases in ESPs play vital roles in disrupting gut epithelial integrity and mediating IIL invasion, growth and survival in hosts, and aspartic proteases and metalloproteases also participate in larval invasion and development. The results suggest that serine proteases and cysteine proteases in *T. spiralis* IIL ESPs have the potential to be regarded as the main candidate target molecules of vaccines against larval invasion and development.

## Abbreviations

AW: adult worm
DAPI: 4′,6-Diamidino-2-phenylindole
DMSO: dimethyl sulfoxide
dpi: days post-infection
EDTA: ethylenediaminetetraacetic acid
ESPs: excretory/secretory proteins
FBS: foetal bovine serum
Fib: fibronectin
hpi: hours post-infection
IEC: intestinal epithelial cell
IIL: intestinal infective larvae
MEM: Modified Eagle’s Medium
ML: muscle larvae
NBL: newborn larvae
pepstatin: pepstatin A
PI: propidium iodide
PMSF: phenylmethylsulfonyl fluoride
PVDF: polyvinylidene fluoride
RT: room temperature
TBST: Tris-buffered saline containing Tween
TJs: tight junctions
1,10-Phe: 1,10-Phenanthrolin
